# Correction: Human Cystathionine-β-Synthase Phosphorylation on Serine227 Modulates Hydrogen Sulfide Production in Human Urothelium

**DOI:** 10.1371/journal.pone.0141027

**Published:** 2015-10-15

**Authors:** Roberta d’Emmanuele di Villa Bianca, Emma Mitidieri, Davide Esposito, Erminia Donnarumma, Annapina Russo, Ferdinando Fusco, Angela Ianaro, Vincenzo Mirone, Giuseppe Cirino, Giulia Russo, Raffaella Sorrentino

The fourth author’s name is spelled incorrectly. The correct name is: Erminia Donnarumma. The correct citation is: d’Emmanuele di Villa Bianca R, Mitidieri E, Esposito D, Donnarumma E, Russo A, Fusco F, et al. (2015) Human Cystathionine-β-Synthase Phosphorylation on Serine227 Modulates Hydrogen Sulfide Production in Human Urothelium. PLoS ONE 10(9): e0136859. doi:10.1371/journal.pone.0136859
